# Editorial: Molecular and cellular interactions of myelin in neurodevelopmental & neurodegenerative disorders

**DOI:** 10.3389/fncel.2023.1359184

**Published:** 2024-01-11

**Authors:** Paschalis Theotokis, Lida Zoupi, Marie-Ève Tremblay, Jing-Wei Zhao

**Affiliations:** ^1^2nd Department of Neurology, University General Hospital of Thessaloniki AHEPA, Thessaloniki, Greece; ^2^Centre for Discovery Brain Sciences, University of Edinburgh, Edinburgh, United Kingdom; ^3^Simons Initiative for the Developing Brain, University of Edinburgh, Edinburgh, United Kingdom; ^4^Division of Medical Sciences, University of Victoria, Lekwungen and WSÁNEĆ Traditional Territories, Victoria, BC, Canada; ^5^Institute of Neuroscience, Zhejiang University School of Medicine, Hangzhou, China

**Keywords:** myelin, neurodevelopment, neurodegenerative disorders, multiple sclerosis, caloric mimetics, peripheral nerve alterations, diagnostic tools, neurorepair

Myelination is a complex process linked to the functional maturation and plasticity of the central nervous system (CNS). Orchestrated by oligodendrocytes, which responsively adapt to environmental cues, their interactions with surrounding neurons and non-neuronal cells shape the intricate functioning of neuronal circuits. Myelination begins prenatally, continues through adolescence and well into adulthood, regulating adult executive and cognitive functions while contributing to cell senescence during aging in both CNS and peripheral nervous system (PNS). Damage to the myelin sheath impairs signal transmission, often leaving the denuded axon prone to further damage and degeneration. As such, in many demyelinating diseases, myelin loss is concurrent with synaptic loss and neuronal damage while at the same time, several neurodegenerative disorders display profound dysmyelination underscoring the indispensable role of myelin in preserving healthy brain networks.

This Research Topic aims to provide a comprehensive update and discussion on novel findings, highlighting the molecular and cellular interactions of myelin in neurodevelopmental and neurodegenerative disorders. Special emphasis was placed on myelin therapeutics, including inhibitors and caloric mimetics, along with innovative imaging techniques. More precisely, this Research Topic integrates four distinct studies, each offering a unique perspective to enhance our comprehension of myelin dynamics and its implications across conditions, ranging from multiple sclerosis (MS) to age-related peripheral nerve alterations.

Myelin is the primary component of oligodendrocytes, yet it is significantly influenced by neuronal and, notably, non-neuronal glial cells (Plemel et al., 2023). Among these, microglia's role has consistently taken precedence, possibly owing to their intriguing origin (Dermitzakis et al., 2023a,b). However, it is crucial to acknowledge that myelinogenesis is also propelled by a distinct yet substantial array of cues (Dermitzakis et al., 2022). One of the latest trends in manipulating glial cells involves the utilization of a ketogenic diet (González Ibáñez et al., 2023) and caloric restriction. In this Research Topic, the work of Kaplanis et al. explores the effects of nicotinamide (NAM), a caloric restriction mimetic, on myelin production under demyelinating conditions. From *ex vivo* models to *in vivo* experiments, NAM showcases its potential to enhance myelination and remyelination, through its effect on microglia and astrocytes, delineating the requirement of a less inflammatory environment that fosters the remyelination process in addition to the effect that elevating NAD+ level enhances the differentiation of the aged OPC (Ma et al., 2022).

The investigation into the aging process has become a contemporary focus of scientific inquiry, reflective of the current research landscape (Li et al., 2021). Impaired metabolism of oligodendrocyte progenitor cells and axons in demyelinated lesions and in the aged CNS has already been reported (Zhao et al., 2022). Helbing et al. lead us through an exploration of the aging PNS, unveiling the complex interplay of myelin maintenance, degradation, and clearance. Contrary to expectations, the myelin proteome remains relatively stable during mouse aging, with only subtle changes observed. This study lays the foundation for future investigations, providing a valuable dataset and resource for understanding peripheral nerve myelin in the context of aging.

On the contrary, in the landscape of CNS diseases, particularly with the high prevalence of MS, the exploration of modulators to address its progressive nature is a focal point of rigorous investigation. Within our Research Topic, Rashidbenam et al. delves into the repercussions of myelin debris accumulation on neurorepair, underscoring the crucial role of myelin-associated inhibitory factors (MAIFs) as potential therapeutic targets. The spotlight is on Nogo-A, a principal MAIF extensively studied in MS models such as experimental autoimmune encephalomyelitis (Theotokis et al., 2012; Theotokis and Grigoriadis, 2018), and its cognate receptor NgR, offering a promising avenue for fostering neurorepair during progressive MS. More specifically, investigations of NgR in plaque modification using the mononuclear system and successful trajectories in stem cell transplantation with neural precursor cells (Theotokis et al., 2022), contribute to this evolving narrative. It is crucial nonetheless, for meaningful therapeutic approaches to demyelinating diseases, to consider selective vulnerability, asking whether neurodegeneration in MS affects specific neuronal components and if it results from demyelination, highlighting a potential link between these two (Schirmer et al., 2019; Zoupi et al., 2021).

Last but certainly not least, a crucial addition to this Research Topic is the clinically relevant identification in Myelin Water Imaging (MWI). In the landscape of myelin alterations, various techniques have been devised to specifically assess these changes, with the myelin water fraction (MWF) emerging as a key proxy to measure the health of myelinated tracts. Mohammadi et al. introduces a novel MWI technique, the STAIR-EPI sequence, which combines the efficacy of the short TR adiabatic inversion recovery (STAIR) sequence with echo-planar imaging (EPI), proving to be robust in detecting myelin loss in both MS lesions and normal-appearing white matter. The findings promise enhanced diagnostic capabilities, marking a significant stride in our ability to quantify myelin alterations in neurological disorders.

In conclusion, the examinations undertaken in these four articles illuminate the complex landscape of myelin throughout life and in demyelinating and neurodegenerative disorders. From molecular intricacies and caloric restriction mimetics to the stability of the myelin proteome during aging and the clinical potential of advanced imaging techniques, these studies collectively contribute to a deeper understanding of myelin dynamics. As we navigate the myelin frontier, these insights pave the way for innovative therapeutic approaches and diagnostic tools, offering new hope in the pursuit of effective interventions for neurological disorders.

## Author contributions

PT: Writing – original draft. LZ: Writing – review & editing. M-ÈT: Conceptualization, Supervision, Writing – review & editing. J-WZ: Investigation, Validation, Writing – review & editing.
